# Assessment of Well-Being and its determinants Among University Staff

**DOI:** 10.1192/j.eurpsy.2025.2013

**Published:** 2025-08-26

**Authors:** A. Abbes, I. Sellami, A. Feki, M. Hajjaji, S. Baklouti, M. L. Masmoudi, K. Jmal Hammami

**Affiliations:** 1 Occupationnal medecine; 2 Rheumatology, CHU Hedi Chaker, Sfax, Tunisia

## Abstract

**Introduction:**

Well-being enables people to cope with life’s stressors and to contribute well to community life. It is associated with better productivity in the workplace.

**Objectives:**

Our study aims to assess well-being and its determinants among university staff.

**Methods:**

We conducted a descriptive, analytical and cross-sectional survey to assess well-being and its determinants among university staff. The survey was carried out during a one-day training session on mental health promotion using a two-part questionnaire. The first part assessed socio-professional characteristics. The work ability was assessed using the Work Ability Score (WAS) questionnaire. Perceived physical workload was assessed by the Borg score. The second part assessed participants’ well-being using the Mental Health Continum Short Form (MHC-SF) questionnaire.

**Results:**

Our population comprised 65 participants, 67.7% of whom were female. The mean of perceived physical workload was 4.7±1.5. The job satisfaction mean was 3.8±1.3. We found that 56.9% of our participants (n=37) had good to excellent work ability and 43.1% rated their work ability as poor to moderate. The mean MHC-SF score was 41.2±16.1. The mean scores for emotional, psychological and social well-being were 8.4±4.2, 19.9±7.2 and 12.8±6.3 respectively. Sixty percent of participants reported languishing to moderate mental health and 40% were flourishing. Bivariate analysis showed that participants’ wellbeing was associated with high work ability and high perceived physical workload.

**Conclusions:**

Well-being among university staff deserves to be studied given its impact on work performance. Actions to promote well-being among these staff are urgently needed not only to improve the well-being and productivity of this population but also to prevent a negative influence on students.

**Disclosure of Interest:**

None Declared

